# Quantifying the variability between multiple multiplanar reconstructions of computed tomography scans

**DOI:** 10.1186/s42490-021-00047-7

**Published:** 2021-02-01

**Authors:** James E. Miles, Lene E. Buelund

**Affiliations:** Department of Veterinary Clinical Sciences, Faculty of Health and Medical Sciences, University of Copenhagen, Dyrlægevej 16, 1870 Frederiksberg C, Denmark

**Keywords:** Computed tomography, Reconstruction, Skeletal, Alignment, Variability

## Abstract

**Background:**

Multiplanar reconstructions of computed tomography (CT) scans can alleviate issues with bone or joint positioning during scan acquisition. The repeatability of these reconstructions is dependent on human operators applying reconstruction criteria, and therefore is subject to error, which could affect measurement reliability for angular or spatial measurements made for orthopaedic surgery.

We describe a method for quantifying inter-reconstruction variability numerically and graphically using metadata from the CT header to find vectors describing reconstruction axis alignment. The approach is demonstrated using 3 sets of computed tomographic reconstructions of 24 vulpine femorotibial joints.

**Results:**

Vectors describing axis alignments permitted identification and subsequent analysis of deviations from optimal alignment between reconstruction sets. For the worked example, alignment deviations equivalent to femoral abduction/adduction were nearly twice those for extension/flexion, and simulation of the effects of these deviations on measurements closely matched published data.

**Conclusions:**

The method presented here is straightforward and permits numerical and graphical analysis of reconstruction variability. Reconstruction alignment variability should be considered before adopting new reconstruction criteria for clinical use, and evaluated whenever there is suspicion that reconstruction variability could unduly influence subsequent measurements. These evaluations may help drive improvements in reconstruction criteria. The methods described here could also be employed for comparing patient positioning between scans and between different scan modalities.

## Background

Ideally, computed tomographic (CT) scans of a patient’s bones and joints should be obtained with the region of interest optimally aligned with the gantry. Optimal alignment can be difficult to achieve in distinct human medical populations [1–3]. Similar issues are faced in the veterinary field, where CT is becoming increasingly popular for evaluation of orthopaedic disease, because of anatomic constraints on limb positioning. Veterinary applications include measurement of femoral anteversion (torsion) [4], femoral varus [4, 5], tibial torsion [4], tibial tuberosity alignment [4, 6], and tibial tuberosity-trochlear groove (TT-TG) offset distance [7]. Diagnostic measurements in veterinary orthopaedics frequently necessitate multiplanar reformatting of the native scans because adequate alignment of the patient’s bones or joints with the gantry can be difficult or impossible to achieve.

Limited data on reconstruction variability are available. In humans, positional variability does not have a consistent effect, with tibial torsion measurements reportedly resilient [8] and TT-TG measurements quite sensitive [9]. The poor reported agreement between CT and MRI scanning for TT-TG [10, 11] and between repeated CT measurements of TT-TG [12] could in part be due to positional variability between scans. These issues are likely similar for veterinary uses of CT. A single observer experimental investigation into inter-reconstruction repeatability of TT-TG measurements made in the red fox (*Vulpes vulpes*) found a marked increase (1.6 mm vs 0.5–0.7 mm) in the repeatability coefficient for measurements made in the *xy* plane between reconstructions as opposed to within reconstructions [7]. The repeatability coefficient is an estimate below which the maximum absolute difference between two paired measurements should lie in 95% of instances [13]. If reconstruction variability were negligible, repeatability coefficient values would be expected to be similar in all cases: the observed discrepancy suggests that reconstructions were not identical and that these differences impacted subsequent measurements.

In order to reduce measurement variation between reconstructions or scans at different time points, better reconstruction or positioning criteria could be used. However, this presupposes a technique to evaluate reconstruction or inter-scan variability and thus compare the effects of different criteria on the repeatability of multiplanar reconstructions or patient positioning. Multiplanar reconstructions are made by rotations of the native scan about the *x*, *y* and *z* axes. Information about the final orientation of the reconstruction relative to the original axes is stored in the DICOM (Digital Imaging and Communications in Medicine) header for the reconstructed images as a series of directional cosines which describe the orientation of the first row and first column of each reconstruction slice [14].

Although reconstruction variability appears initially to be a three-dimensional problem, errors relating to rotation about one axis (the viewing axis) can usually be disregarded because measurements are typically made in planes normal to this axis, either on a single slice or by projection of points from one slice onto another. For any set of reconstruction criteria and a particular measurement, the issue of reconstruction variability may therefore be reduced to consideration of rotational errors about the other two axes, which together define the measurement plane.

A method has been described using the separation angle to quantify reconstruction variability, but this has the disadvantage of combining errors in rotation about two axes into a single angular description [7]. This leads to an inevitable loss of information and hampers efforts to identify the underlying causes of reconstruction variability and thus ameliorate them.

This paper presents an improved method to quantify reconstruction variability in multiplanar reformatted CT scans, and is intended as an aid to researchers in investigating reconstruction variability and developing improved reconstruction criteria.

## Results

**Fig. 1:**
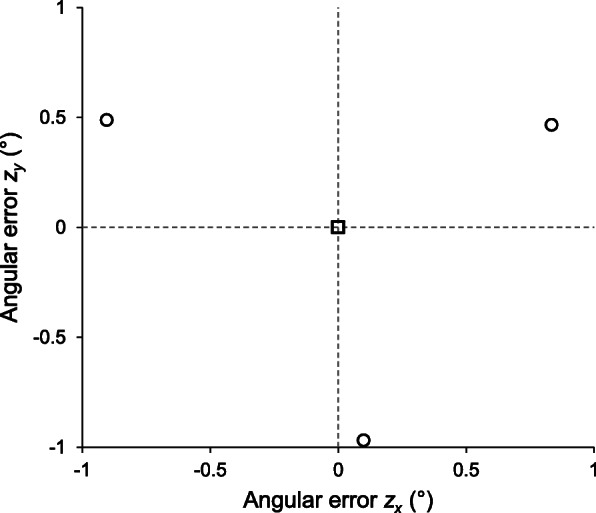
Orthographic plot of selected data. The three circles represent the projections of the angular deviations of the *z* axis between the three reconstructions along the x and y axes compared to the true alignment of the *z* axis at the origin. The centroid (square) of the three reconstructions is located at the origin. Imprecision during multiplanar reconstruction of the native scans has produced a ‘wandering’ projection of the *z* axis about this centroid

For the three reconstructions of the single femorotibial joint shown in the worked example section, the directional cosines of interest for the *z*-axis, along with the equivalent angular deviations from the *z*-axis in the directions of the *x* and *y* axes are shown in Table 1. A stereographic plot of the three points is shown in Fig. 1.

**Table 1:** Cosines of interest and their associated angles for the *z* axis

Recon	*z*_*x*_ cosine	*z*_*x*_ angle (°)	*z*_*y*_ cosine	*z*_*y*_ angle (°)
1	−0.015	0.835	−0.008	0.465
2	0.016	−0.905	−0.009	0.488
3	−0.002	0.100	0.017	−0.969

When considering *z* axis deviations from perfect alignment, errors along the *x* and *y* axes are of interest. The cosines produced by reverse rotation are shown for the three reconstructions (recon) along with their associated angular deviations from the *z* axis (corrected by subtraction of 90°)

**Fig. 2:**
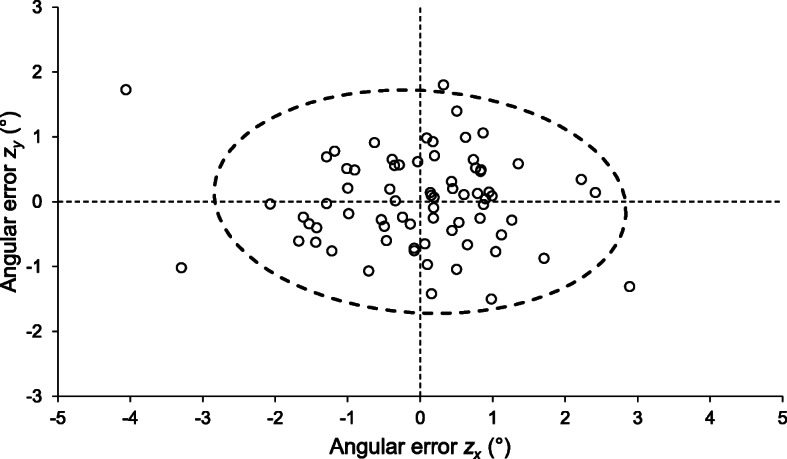
*Z*-axis alignment errors between reconstructions for 24 native scans of the femorotibial joint. Errors along the *x* axis represent rotations about the *y* axis, and vice versa. Angular errors have been derived from the axis cosines and corrected by subtraction of 90°. The 95% error ellipse is shown overlying the data points, rotated clockwise by the ellipse angle 4.4°. There is increased variability in rotation about the *y* axis (equivalent to femoral abduction/adduction) compared to the *x* axis (equivalent to femoral extension/flexion), as evidenced by flattening of the ellipse and the underlying data spread

When this process was continued for all 24 femorotibial joints, variances ${\sigma}_{z_x}^2$ and ${\sigma}_{z_y}^2$ were 1.35 deg^2^ and 0.49 deg^2^ respectively. Covariance was − 0.07 deg^2^, yielding an ellipse angle of − 4.4° (using Eq. 5). After scaling the output of Eq. 4 for a 95% error ellipse (*k* = 2.448), the semi-major (along the *x* axis) and semi-minor (along the *y* axis) axes were 2.8° and 1.7°, respectively. The 95% error ellipse area was 15.3 deg^2^. An orthographic plot of these data and the associated ellipse is shown in Fig. 2.

The full set of direction cosines and associated data is available, demonstrating application of steps 5–9 and 10–14 described above [15].

### Extrapolation to TT-TG study

The mean TT-TG measurement was 0.23 mm medial to the orientation of the trochlear sulcus, with a range of 1.9 mm, corresponding to an error due to reconstruction variability of ±0.95 mm. This range was essentially unaltered by zeroing the tilt semi-axis (simulating no rotation about the *x*-axis): in contrast, zeroing the spin semi-axis to zero (simulating no rotation about the *y*-axis) reduced this range to 0.1 mm.

The magnitude of the z_x_ angle, the major contributor to inter-reconstruction variability, was positively and significantly correlated with the inter-reconstruction variance in TT-TG measurements obtained previously [7], Spearman’s rho 0.53 (*p* < 0.001).

## Discussion

The method for quantifying inter-reconstruction variability demonstrated here is straightforward, especially with the aid of a spreadsheet, and has the advantage over the only previously described method [7] of not reducing two variables to a single variable. The ellipse parameters and graphical analysis enable ready appreciation of differences in axis alignment between reconstructions made from the same native scans. The method described here could readily be adapted for evaluation of positional variability between native scans obtained at multiple time points, or positional variability between scan modalities, both of which may influence subsequent measurement repeatability [10–12].

We are not aware of previous reports focusing on inter-reconstruction variability. Possible reasons include belief that inter-reconstruction variability or its effects are insignificant, or a lack of appreciation that inter-reconstruction variability can occur or can be quantified. Although some indication of the effect of reconstruction variability can be obtained by comparing measurements from different reconstructions, if these are performed by the same observer there may be learning bias in the obtained measurements, and if performed by different observers it is impossible to distinguish between the effects of reconstruction and observer. It is important to be able to determine at which level measurement differences arise (i.e. during multiplanar reconstruction or during reading), since this will alter the approaches needed to minimize these differences. If differences are mostly due to reconstruction variability, optimizing reconstruction criteria and focusing training here is more relevant than focusing on landmark identification for the actual measurements, and vice versa.

We recommend a combined graphical and numerical evaluation of angular error data, in order to exclude extreme outliers that may result in ellipse parameter bias and to fully appreciate the data. Graphical analysis allows rapid appreciation of areas of concern, for example marked deviations along one axis or covariation of the data. Numerical analysis provides data which may be used to compare with future results obtained using improved reconstruction criteria or different methods. The choice of error ellipse size is left to the individual researcher, although the 95% error boundary is a generally accepted limit. The approach used here assumes random variability and normal distribution. The accuracy of the estimates for the ellipse axes (equivalent to the standard deviation) is therefore dependent on the chi-squared distribution with a degree of freedom equal to *nm*-1, where *n* is the number of individuals or samples and *m* is the number of reconstructions per individual or sample [16]. Using 3 reconstructions per limb as shown here reduced the range of the 95% confidence interval for the ellipse axes by a factor of 0.8 compared to using only 2 reconstructions: using 4 would further reduce the range by a factor of 0.9. Individual researchers will need to determine their accuracy requirements in relation to the extra workload involved in creating additional reconstructions and the number of individuals or samples available.

The data presented here are orthographically projected. In an orthographic projection, points on the spherical surface are projected onto the associated plane along lines normal to this plane. With increasing angular deviation of these points from the axis connecting the pole in contact with the plane to the antipodean pole, the orthographic projection underestimates the distances from the contact pole to the points. In contrast, a stereoscopic projection, in which points are projected along a line from the antipodean pole passing through the points and onto the projection plane, results in slight overestimation. However, at the angles at which researchers are likely to be investigating reconstruction variability, these errors are likely to be insignificant.

In the example shown, the data variance along the *x* axis was almost three times that along the *y* axis, indicating some difficulties in precisely controlling rotation about the *y* axis during multiplanar reconstruction of the native scans. This rotation can be considered equivalent to abduction and adduction of the femur.

Graphical analysis showed most reconstructions were within 3°-4° of the intended neutral alignment along the x-axis. This is not dissimilar to reported deviations from intended alignment with the CT gantry for human femora and knee joints of between 1°-11° of adduction [2, 9]. Ultimately, the significance of this variability will be decided by the clinician or surgeon in light of its effect on subsequent measurements. Reconstructions in the red fox TT-TG study were performed to strict criteria and double checked using maximum intensity projection images [7], but these precautions were inadequate to completely constrain either positional or measurement variability.

Additional safeguards on reconstruction variability, a change in reconstruction criteria or a change in measurement technique would appear necessary to reduce measurement variability. A major problem with this series of reconstructions was the use of landmarks solely confined to the trochlea to define the sagittal plane, in contrast with typical medical practice. This was a deliberate choice to test a hypothesis that the tibial tuberosity is neutrally aligned with the trochlea in the canid femorotibial joint (i.e. that the local TT-TG value is zero). In contrast, the coronal plane was defined by landmarks at either end of the femoral diaphysis, which markedly constrained tilt variation. Whilst use of similarly placed landmarks for defining the sagittal plane would have likely constrained y-axis rotation similarly, values for TT-TG measurement would then become dependent on distal femoral varus or valgus. This would complicate interpretation and require subsequent correction for femoral morphology, and was the reason for restricting the landmarks so severely for these reconstructions.

Simulated rotation and projection of coordinates retrieved from the TT-TG study CT scans confirmed the findings of the graphical analysis, and provided additional information. Reconstruction variability alone could produce TT-TG measurement errors of up to ±0.95 mm, based on a 95% error ellipse, and this was mostly attributable to *spin* errors, or rotation about the *y*-axis. In the actual TT-TG study [7], the inter-reconstruction repeatability coefficient (representing the maximum expected difference between 95% of paired observations) was ±1.6 mm, which fits very well with the predictions from the simulation, given intra-reconstruction repeatability coefficients of ±0.5–0.7 mm (as reconstruction variability of 0.95 + intra-reconstruction variability of 0.5–0.7 = inter-reconstruction variability of 1.45–1.65). Here we assume the intra-reconstruction values to represent solely observer error, and the inter-reconstruction values to represent the sum of observer error and reconstruction variability. The magnitude of the *spin* error (z_x_) was positively correlated with inter-reconstruction variability, supporting the importance of controlling reconstruction variability in order to minimise measurement error. In this specific instance, use of the approach outlined here in combination with the simulation would likely have resulted in further efforts to reduce inter-reconstruction variability prior to starting the measurement process.

The relevance of similar error magnitudes will vary with the type of measurement, landmarks involved, and patient population. In one study in humans, a mean femoral adduction of 6.6° resulted in mean femoral anteversion measurements over 4° greater than with neutral alignment [2]. Clinically, patient measurements are often compared to pre-determined cut-off values in order to determine the need for surgical intervention, and the degree of correction required. If similar variability occurred in CT scans of dogs with medial patellar luxation, for example, it is clear that mis-classification as either requiring or not requiring femoral detorsion could occur, along with the potential for over- or under-correction of abnormal anteversion. The researcher may approach this problem in one of two ways. Where there is pre-existing literature regarding the effect of alignment on measurement error, likely thresholds for reconstruction error may be directly inferred and used to compare with the error ellipse obtained above. Where such information does not exist, a trial and error approach may be employed using the described macro [17], to find input limits at which the simulated measurement error becomes unacceptably large. These input limits may then be used in place of literature-derived values.

## Methods

### Principals

The method outlined below is relatively straightforward and can be accomplished with the aid of a spreadsheet. A workbook containing spreadsheets to perform steps 5–9 described below is freely available [18].

### Extracting directional cosines from the DICOM header

The initial steps require calculation of the rotations performed during multiplanar reconstruction and can be summarised as:

1. Acquire native scans of the region of interest from multiple individuals.
2. Perform multiplanar reconstruction according to a set of defined criteria.
3. Repeat step 2 at least twice so that for each individual there are at least 3 versions of the multiplanar reconstruction for analysis.
4. Group these reconstructions in sets, within which each individual features only once.
5. For each reconstruction in each set, extract the DICOM header data from the Image Orientation (0020, 0037) tag. This may be achieved using the DICOM header viewer available in most viewing software or in bulk using a macro in ImageJ [19], available in the aforementioned workbook. The Image Orientation tag records the direction cosines for the first row and first column of each image slice in the stack, corresponding to unit vectors along the *x*, *y* axes.
6. Calculate the direction cosines for the *z* axis (see Table 2). This can be accomplished directly using in the aforementioned workbook, which both converts the text output of the macro into individual direction cosines and also calculates the *z* axis components.

**Table 2:** Calculation of directional cosines

Axis component	Primary axis		
	*x*	*y*	*z*
*x*	*x*_*x*_	*y*_*x*_	*z*_*x*_ *= x*_*y*_*y*_*z*_*-x*_*z*_*y*_*y*_
*y*	*x*_*y*_	*y*_*y*_	*z*_*y*_ *= x*_*z*_*y*_*x*_*-x*_*x*_*y*_*z*_
*z*	*x*_*z*_	*y*_*z*_	*z*_*z*_ *= x*_*x*_*y*_*y*_*-x*_*y*_*y*_*x*_

The DICOM header tag Image Orientation (0020, 0037) contains directional cosines describing unit vectors along the first row (*x* axis) and first column (*y* axis) of each reconstruction slice relative to the native scan. Since the *z* axis is orthogonal to the *x* and *y* axes, the directional cosines for this axis can be calculated as shown

For an investigation of positional variability between native scans at different time points, steps 1–3 may be achieved by acquisition of these scans for multiple patients.

### Defining the rotational matrix and its inverse

Each set of components for each axis (e.g. *x*_*x*_, *x*_*y*_, *x*_*z*_) represents a unit vector describing the orientation of that axis. Since the three axes are orthogonal, their vector components, taken together, represent an orthogonal rotation matrix **R**:

1 $$ \mathbf{R}=\left(\begin{array}{ccc}{x}_x& {y}_x& {z}_x\\ {}{x}_y& {y}_y& {z}_y\\ {}{x}_z& {y}_z& {z}_z\end{array}\right). $$

Matrix **R** describes the transformation from the initial patient orientation to the reconstruction orientation. The transpose of this matrix, **R**^**− 1**^, enables back rotation from the reconstruction axes to the original orientations of the native scan. Because initial patient orientation will differ between native scans, this source of variation needs to be removed. This can be achieved by finding mean vectors for each individual across reconstructions, deriving **R**^**− 1**^ from these mean vectors, and then rotating the separate vectors for each reconstruction. These steps can be performed automatically in the spreadsheet PrecisionCalculator within the PrecisionAnalyser workbook.

7. The mean vectors for each individual across their reconstructions are found by averaging each set of axis components, e.g.

2 $$ {\overline{x}}_x=\frac{1}{n}\left({x}_{x_1}+{x}_{x_2}+..+{x}_{x_n}\right). $$

8. The back rotation matrix **R**^**− 1**^ for each individual is found by arranging the mean vector components in rows to form a 3 × 3 matrix, such that

3 $$ {\mathbf{R}}^{-\mathbf{1}}=\left(\begin{array}{ccc}{\overline{x}}_x& {\overline{x}}_y& {\overline{x}}_z\\ {}{\overline{y}}_x& {\overline{y}}_y& {\overline{y}}_z\\ {}{\overline{z}}_x& {\overline{z}}_y& {\overline{z}}_z\end{array}\right). $$

9. Multiply each set of vectors for each reconstruction by **R**^**− 1**^ to centre the **x**, **y**, **z** vectors on their respective primary axes, removing variation due to initial positioning.

### Identifying the vectors of interest

The primary axis of interest (viewing axis) must now be identified. The distribution of vectors around the viewing axis represents the misalignments of this axis introduced during multiplanar reconstruction. For measurements made in the sagittal, coronal and transverse planes, the primary axes of interest will typically be the *x*, *y*, and *z* axes respectively. Of the three components defining this primary axis of interest, the two representing deviations along the orthogonal axes should be retrieved. For example, if the measurement of concern is obtained from transverse slices, the primary or viewing axis will be *z*, and the two orthogonal axes for which data should be retrieved will be *z*_*x*_ and *z*_*y*_. The distribution of vectors along *z*_*x*_ represents the effects of rotation of the viewing axis about the *y*-axis, whereas the distribution along *z*_*y*_ represents rotation of the viewing axis about the *x*-axis, as will be seen later.

### Graphical and numerical evaluation

These components can be converted into angular deviations along the two orthogonal axes by finding the arccosine of each component of interest before plotting using an orthographic projection. As shown in Table 3, at restricted angular deviations likely to be experienced in practice there is little difference between true arc length along the surface of the unit sphere and the orthographic projection. It should be noted that deviations along one axis represent rotational error about the perpendicular axis.

**Table 3:** Comparison of arc length and the orthographic projection

Angle (°)	Arc length	Orthographic distance	Underestimate (%)
0	0.000	0.000	0.00
1	0.017	0.017	− 0.01
2	0.035	0.035	−0.02
3	0.052	0.052	−0.05
4	0.070	0.070	−0.08
5	0.087	0.087	−0.13
6	0.105	0.105	−0.18
7	0.122	0.122	−0.25
8	0.140	0.139	−0.32
9	0.157	0.156	−0.41
10	0.175	0.174	−0.51
15	0.262	0.259	−1.14
20	0.349	0.342	−2.02

The vectors found in the study define points on the surface of a unit sphere which must be projected onto a plane for graphical analysis. The amount of error introduced by the orthographic projection relative to the true distance between the polar contact point between the sphere and the plane and another point on its surface is shown for angular deviations from the axis normal to the plane. Typical angular deviations reported in the literature for the femur in humans are 1°-11° [2, 9]

The data can now be analysed using Model II regression techniques [20] to find ellipse axes and related characteristics describing the distribution of the points.

10. Find the variances (${\sigma}_a^2,{\sigma}_b^2$) for the components of interest (e.g. *x*_*y*_, *x*_*z*_) across all reconstructions, and the Pearson correlation coefficient *ρ* between these components.
11. Calculate the covariance as *σ*_*ab*_ = *ρ* ∙ *σ*_*a*_ ∙ *σ*_*b*_ and derive the eigenvalues of the variance-covariance matrix $$ \left(\begin{array}{cc}{\sigma}_a^2& {\sigma}_{ab}\\ {}{\sigma}_{ab}& {\sigma}_b^2\end{array}\right) $$ using the formula

4 $$ \lambda =\frac{\sigma_a^2+{\sigma}_b^2\pm \sqrt{{\left({\sigma}_a^2+{\sigma}_b^2\right)}^2-4\left({\sigma}_a^2\bullet {\sigma}_b^2-{\sigma_{ab}}^2\right)}}{2}. $$

12. The major and minor semi-axes of the error ellipse corresponding to the errors about the two reconstruction axes of interest are found as the square roots of *λ*_*1*_ and *λ*_*2*_ derived using Eq. 4.
13. The ellipse area *A* can be calculated as *A* = *π* ∙ *r*_*a*_ ∙ *r*_*b*_.
14. If there is substantial covariance, the ellipse angle *θ* (representing anticlockwise rotation from the horizontal semi-major axis) can be calculated as

5 $$ \theta =\frac{1}{2}{\tan}^{-1}\left(\frac{2{\sigma}_{ab}}{\sigma_a^2-{\sigma}_b^2}\right). $$

These semi-axis values define a 39% error ellipse: a scaling factor *k* can be used to encompass other error probabilities [21] using $k=\sqrt{-2\ln \left(1-p\right)}$ where 0 < *p* < 1. These parameters enable plotting of the error ellipse, comparison between different reconstruction criteria, and identification of potentially problematic reconstruction axes.

### Worked example

Three sets of multiplanar reformatted CT scans produced for a published study of TT-TG measurement in 24 red foxes (*Vulpes vulpes*) [7] were used as the source material [22]. Cadavers were obtained from a commercial unit following euthanasia by electrocution in accordance with Danish law and were unaffected by orthopaedic disease of the hind limbs based on physical inspection and radiography. Institutional ethical approval was obtained. During scanning of the hind limb, anatomical constraints resulted in obliquity of both femora and tibia relative to the gantry. Three reconstructions were produced for each scanned limb by a single operator according to previously defined criteria [7]. Briefly, one plane passed through the centres of the femoral head and medial condyle and parallel to the caudal aspects of both femoral condyles; the second plane was aligned orthogonally and along the femoral trochlear sulcus; the third plane was aligned co-orthogonally to the first two. Based on the published result showing a 3-fold change in measurement repeatability across reconstructions compared to within reconstructions, there was a concern that reconstruction variability could have played a significant role in measurement variation.

Extracted directional cosines, the calculated z axis cosines and the mean vectors (using Eq. 2) for one individual and three reconstructions are shown in Table 4. These yield the transposed rotation matrix (Eq. 3) for this individual of:

$$ {\mathbf{R}}^{-\mathbf{1}}=\left(\begin{array}{ccc}0.9202& 0.3908& 0.0122\\ {}-0.2912& 0.7059& -0.6453\\ {}-0.2611& 0.5905& 0.7635\end{array}\right). $$

**Table 4:** Derivation of mean vector components

Recon	*x*_*x*_	*x*_*y*_	*x*_*z*_	*y*_*x*_	*y*_*y*_	*y*_*z*_	*z*_*x*_	*z*_*y*_	*z*_*z*_
1	0.9224	0.3845	0.0367	−0.2743	0.7190	−0.6386	−0.2720	0.5790	0.7687
2	0.9243	0.3817	0.0000	−0.2937	0.7110	−0.6389	−0.2440	0.5910	0.7693
3	0.9138	0.4062	0.0000	−0.3057	0.6878	−0.6584	−0.2674	0.6017	0.7527
mean	0.9202	0.3908	0.0122	−0.2912	0.7059	−0.6453	−0.2611	0.5905	0.7635

Selected data from one individual and three reconstructions (recon) of a single native scan are shown. The *x* axis and *y* axis data were retrieved from the DICOM header and the *z* axis cosines were calculated as shown in Table 2. The mean vectors were calculated by averaging each component of each axis vector

This matrix is then applied to the individual axis vectors: as an example, the reverse rotation of the z-axis vector from the first reconstruction for this individual is shown.

$$ {\mathbf{R}}^{-\mathbf{1}}\left(\begin{array}{c}{z}_{x_1}\\ {}{z}_{y_1}\\ {}{z}_{z_1}\end{array}\right)=\left(\begin{array}{c}0.9202\bullet -0.2720+0.3908\bullet 0.5790+0.0122\bullet 0.7687\\ {}-0.2912\bullet -0.2720+0.7059\bullet 0.5790-0.6453\bullet 0.7687\\ {}-0.2611\bullet -0.2720+0.5905\bullet 0.5790+0.7635\bullet 0.7687\end{array}\right)=\left(\begin{array}{c}-0.0146\\ {}-0.0081\\ {}0.9997\end{array}\right) $$

### Extrapolation to TT-TG study

To estimate the effect of reconstruction variability on TT-TG measurement accuracy in the initial study [7], a further analysis was performed using a custom Visual Basic for Applications macro in Excel: a workbook containing this macro and guidelines for its use is freely available [17].

Using freely available software (ImageJ, [19]), three dimensional coordinate data representing the locations of the caudal aspects of the lateral and medial femoral condyles, the base of the trochlea sulcus and the tibial tuberosity were transferred to a spreadsheet, along with error ellipse semi-axes (scaled using *k* = 2.45 for a 95% error ellipse) and angle. Running the macro rotated the coordinate data in 200 steps along the ellipse perimeter, projecting the coordinates onto a reference plane and calculating the TT-TG measurement at each step. The TT-TG measurement was calculated as the shortest distance between two lines, both perpendicular to a line passing through the femoral condyle projections, passing through either the trochlear sulcus or tibial tuberosity projection. Output data included mean, standard deviation, minimum, maximum and range of TT-TG measurement for each reconstruction. Runs were repeated with each of the semi-axes alternately set to zero, to estimate the individual contributions of errors in these directions.

## Conclusion

In conclusion, we present a technique that permits quantification of reconstruction variability, the results of which seem to match measurement variation seen in practice. We suggest that reconstruction variability should be evaluated before adopting reconstruction criteria for clinical use, whenever there is suspicion that reconstruction variability could unduly influence subsequent measurements. These evaluations may help drive improvements in reconstruction criteria, permit comparisons between different reconstruction criteria or methods, and can form the basis of geometric modelling of measurement errors due to reconstruction variability. The methods described here could also be employed for comparing patient positioning between scans and between different scan modalities.

## Data Availability

The datasets generated and analysed during the current study are available in the figshare repository: PrecisionAnalyser worksheet, 10.6084/m9.figshare.1544294, Microsoft Excel worksheet, GNU General Public Licence [18]. PrecisionAnalyser with worked example, 10.6084/m9.figshare.1544295, Microsoft Excel worksheet, GNU General Public Licence [15]. CT reconstructions and extracted header metadata, 10.6084/m9.figshare.3435410, CC-BY licence [22]. CTreconSim – a simulator of reconstruction induced measurement variability, 10.6084/m9.figshare.1337839.v6, Microsoft Excel worksheet, GNU General Public Licence [17].

## Abbreviations

CT: Computed tomography
DICOM: Digital Imaging and Communications in Medicine
MRI: Magnetic resonance imaging
TT-TG: Tibial tuberosity-trochlear groove distance
